# Giant Cell Tumour of the Spine

**DOI:** 10.7759/cureus.71166

**Published:** 2024-10-09

**Authors:** Emir M Zulkharnain, Mohd Hisam Muhamad Ariffin, Jin Aun Tan, Muhammad Amir Afiq Zakaria

**Affiliations:** 1 Orthopaedic and Traumatology, Universiti Kebangsaan Malaysia Medical Centre, Kuala Lumpur, MYS; 2 Spine Surgery, Universiti Kebangsaan Malaysia Medical Centre, Kuala Lumpur, MYS; 3 Department of Orthopaedics and Traumatology, Faculty of Medicine, Pusat Perubatan Universiti Kebangsaan Malaysia, Kuala Lumpur, MYS; 4 Orthopaedics and Traumatology, Universiti Kebangsaan Malaysia Medical Centre, Kuala Lumpur, MYS

**Keywords:** gct, giant cell tumour, giant cell tumour (gct), mobile spine gct, spine, spine gct

## Abstract

Giant cell tumour (GCT) is a benign bone tumour characterised by osteoclastic-like multinucleated cells. This tumour predominantly occurs in adults during the third decade of life, commonly in the distal femur, proximal tibia, distal radius, and sacrum. Treatment options include wide local excision with possible adjuvant therapy depending on tumour size and site. We present a case of an 18-year-old lady with no prior medical history complaining of worsening insidious onset localised back pain and preceding numbness of the bilateral lower limbs, diagnosed with a giant cell tumour of the spine (T11).

## Introduction

Giant cell tumour (GCT) typically develops in the metaphyseal regions of long bones, predominantly affecting individuals aged 20 to 40, with a higher prevalence in females. Its occurrence in the spine is rare, accounting for only 1.4-9.4% of primary spine tumours [1]. Despite being classified as benign, GCTs exhibit local aggressiveness and have the potential to metastasise to the lungs. Spinal GCTs, especially above the sacrum, are relatively rare occurrences [2]. Among all giant cell tumours of the bone, 2.9% occur in the vertebrae above the sacrum, and 2.5% are found in the sacrum [2]. Surgical excision is recommended for spinal GCTs; however, anatomical constraints can present challenges, leading to local recurrence rates ranging from 22.4% to 41.7% [1]. Patients or their legal guardians provided informed consent prior to writing the case report.

## Case presentation

An 18-year-old woman with no prior medical conditions visited our clinic, complaining of persistent back pain that had been worsening. She had also begun to experience occasional numbness in both her lower limbs. At the time of her initial visit, her back pain had intensified significantly, escalating from a pain rating of 2/10 to 7/10. Additionally, she had developed weakness in both of her lower limbs, necessitating the use of a walking stick for mobility. Remarkably, within a span of two weeks after her initial presentation, she had lost all function in her lower limbs and required a wheelchair for mobility. Notably, there were no associated issues with bowel or urinary incontinence.

She was admitted, and upon examination, no fasciculation or muscle wasting in the lower limb muscles was observed. The anal tone was found to be intact, and both the anal wink reflex and voluntary anal contraction were present. The neurological examination findings are described as follows (Table 1), based on the American Spinal Injury Association (ASIA) impairment scale.

**Table 1:** Patient’s ASIA Impairment Scale Chart on admission.

	Right motor	Right sensation	Left motor	Left sensation
C5	5	2	5	2
C6	5	2	5	2
C7	5	2	5	2
C8	5	2	5	2
T1	5	2	5	2
L2	0	0	0	0
L3	0	0	0	0
L4	0	1	0	1
L5	0	1	0	1
S1	0	1	0	1

**Figure 1:**
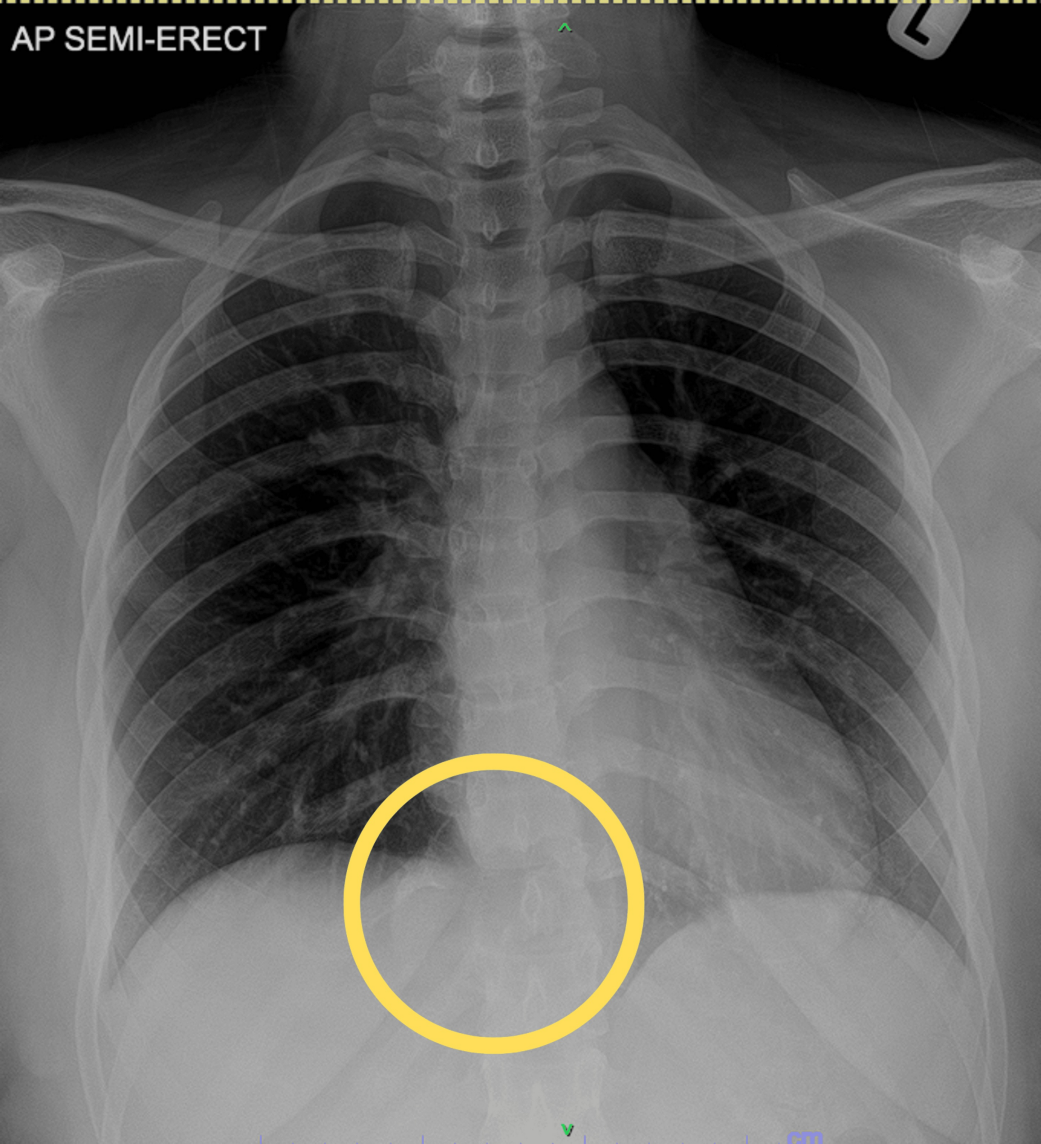
CXR showed suspicious osteolytic lesion arising from T10/T11 that extends right laterally.

**Figure 2:**
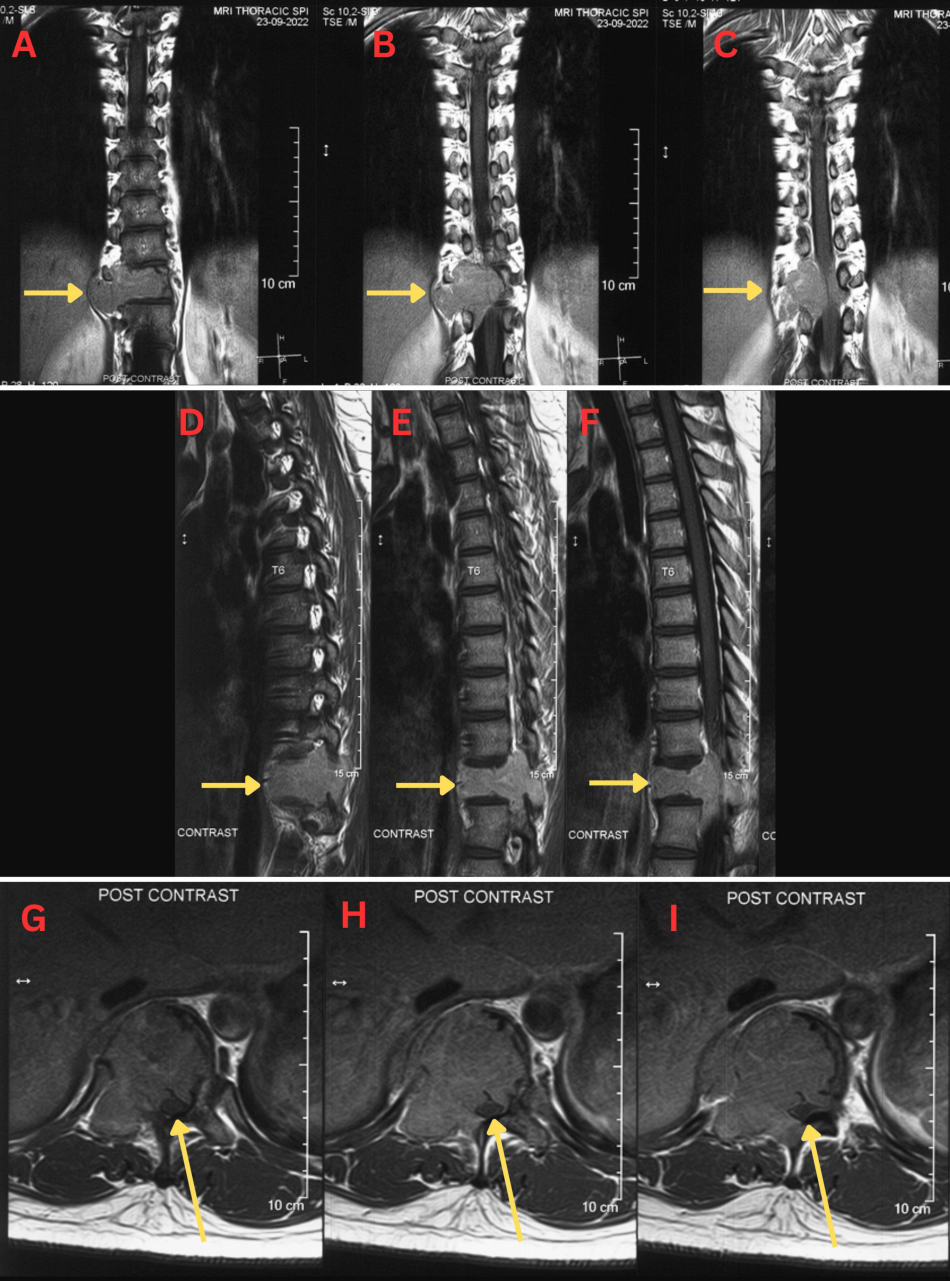
Preoperative T1 post contrast MRI of the thoracic spine in coronal (A,B,C), saggital (D,E,F), and axial (G,H,I) views. The arrows show the lesion on the T11 vertebral body extending towards the right pedicle and posterior element. The mass effect has narrowed the spinal canal and pushed onto the spinal cord, as seen in arrows in the axial (G, H, I) view.

**Figure 3:**
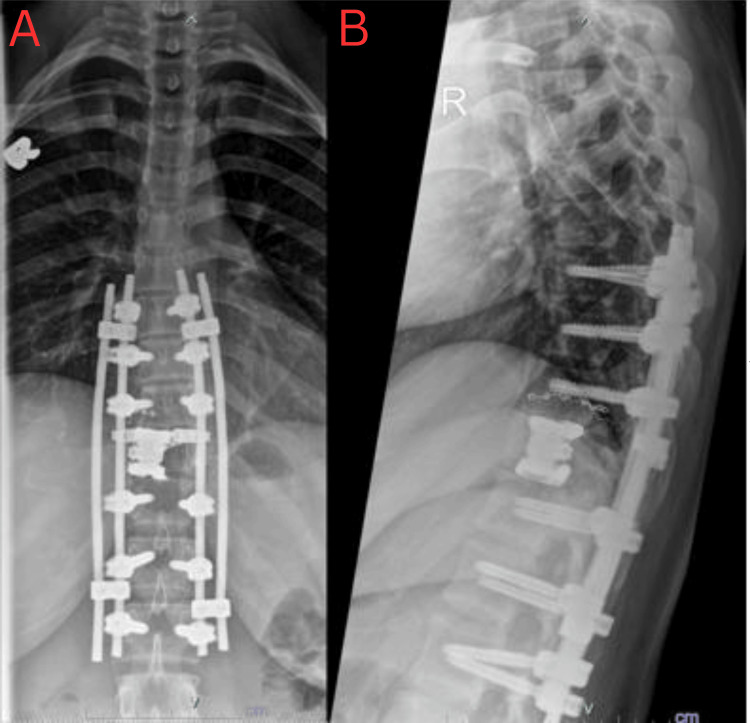
The post-operative thoracic spine X-ray in anteroposterior (A) and lateral (B) views.

**Figure 4:**
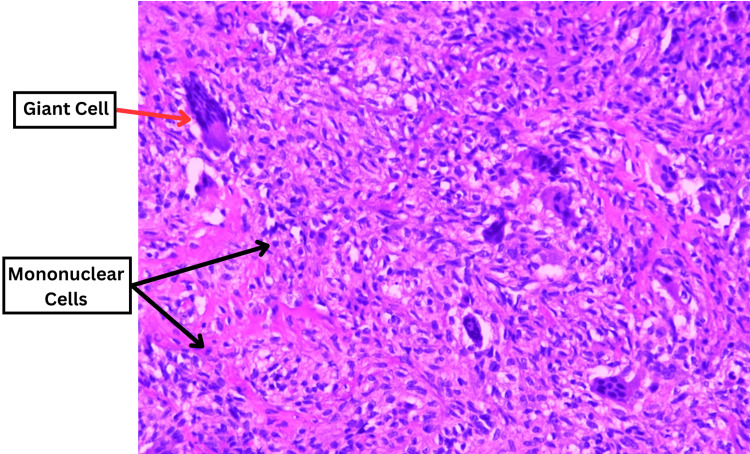
Histopathological image of the tumour composed of proliferating mononuclear cells (black arrows) interspersed with numerous osteoclast-like multinucleated giant cells (red arrow) and histiocytes within fibromatic stroma.

The chest X-ray (CXR) revealed a concerning osteolytic lesion originating from the T10/T11 vertebra, extending predominantly to the right side, with no apparent displacement of the mediastinum (Figure 1). In contrast, the MRI of the entire spine showed an expansive soft tissue mass arising from the T11 vertebral body (measuring 7.5 cm × 5.0 cm × 5.4 cm), extending towards the right pedicle and posterior element (Figure 2). This mass caused a narrowing of the spinal canal, resulting in severe compression of the conus medullaris and the right T11 nerve root. Prior to performing the surgical procedure, an arterial embolisation was carried out at the right intercostal artery supplying the T11 vertebral body, specifically at the T10 level (Figure 3). The surgical intervention involved a T11 corpectomy, removal of the 10th and 11th costovertebral joints, tumour debulking, and posterior instrumentation spanning from T8-T10 and T12-L2 levels. The patient also received intravenous dexamethasone at a dose of 8 mg three times daily for three days to counteract the inflammatory response associated with spinal cord irritation. Histopathological examination of the excised tissue revealed a well-defined tumour characterised by proliferating mononuclear cells forming sheets interspersed with numerous osteoclast-like multinucleated giant cells and histiocytes within a fibrotic stroma (Figure 4). These findings suggest a giant cell tumour originating in the vertebrae.

Following the surgery, the patient underwent 25 cycles of radical radiotherapy at 45 Gy targeting the T10-T12 vertebral region over a span of 4.5 weeks. Remarkably, her neurological symptoms gradually improved, enabling her to regain the ability to walk independently again.

Following surgery, she required the use of crutches due to residual weakness. However, her muscle strength steadily improved over the next three months, eventually returning to full capacity. Additionally, her sensory function fully recovered within one month. The patient has been recurrence-free for two years following her surgery. She has returned to work and is pain-free, able to perform her daily activities just as she did prior to the operation.

## Discussion

A giant cell tumour in the spine is a rare condition in contrast to its more common occurrence in long bones, such as the distal femur, proximal tibia, distal radius, and sacrum [3]. The incidence of GCTs arising from the mobile spine (above the sacrum) ranges from 1.4% to 9.4% and tends to afflict slightly younger patients [4]. Although benign, GCTs can demonstrate local aggressiveness, rendering the clinical management of recurrence of spinal GCTs particularly complex [4].

The clinical presentation of our patient is notable. Initially, she reported localised back pain and numbness in her lower limbs. Over a surprisingly brief period, her condition deteriorated rapidly. These symptoms underscore the gradual onset and aggressive nature of spinal GCTs [1].

The radiological findings in this case were pivotal for diagnosis and treatment planning. The MRI of the entire spine revealed an expansive soft tissue mass arising from the T11 vertebral body, leading to marked compression of the conus medullaris and the right T11 nerve root. This imaging modality enabled precise localisation and tumour assessment for local staging, vital for surgical planning. Though spinal GCTs tend to present as solitary lesions, multicentric cases have been reported [4]. To reduce the local recurrence rate, we proceeded with an en-bloc resection of the affected spinal vertebrae [4].

Spinal GCTs have been reported to be highly vascularised, and performing embolisation prior to surgery has been proven to minimise blood loss and allow a relatively bloodless field of surgery for adequate tumour resection [2]. The embolisation was done for our patient prior to surgery and has limited the blood loss. Some microscopic remnants of the tumour tissue are somewhat expected to be left behind. However, a follow-up of radiotherapy has been recommended to aid in reducing the local recurrence [5], which was also done for our patient. For the tumour resection, we performed posterior instrumentation for the levels T8-T10 and T12-L2 with a metallic cage to replace the T11 for fusion. The usage of corticosteroid for our patient was also vital to reduce oedema and inflammation of the spinal cord caused by the tumour to reduce further irreversible damage. Bisphosphonate use in preventing recurrence has been proven in GCTs on other parts of the body but not the spine [6].

Histopathological examination of the excised tissue is crucial for confirming the diagnosis. In this case, the tumour exhibited proliferating mononuclear cells forming sheets interspersed with numerous osteoclast-like multinucleated giant cells and histiocytes within a fibrotic stroma. These findings were consistent with a giant cell tumour [7].

The post-operative management and patient outcomes are encouraging. The patient underwent both surgery and radiotherapy, resulting in a gradual improvement in neurological symptoms. This case illustrates the importance of a multidisciplinary approach to the management of spinal GCTs involving neurosurgery, orthopaedics, radiology, and oncology.

Close monitoring of the patient’s long-term condition has been proven and recommended to detect early local recurrences [3]. The recommendation is for clinical and radiographic surveillance every three months for the first two years, spaced to every six months for the following three years, and eventually yearly until the 10th year [3]. A yearly CT scan of the lungs is also recommended to detect lung metastases if they do occur [3] due to findings of the average GCT taking 18-24 months to be detected in the lungs as metastases [2].

## Conclusions

In conclusion, spinal GCTs, though rare, present complex challenges due to their aggressive nature. This case highlights the importance of timely diagnosis, with MRI crucial for accurate tumour staging and guiding treatment. The combined approach of en bloc resection, preoperative embolisation, radiotherapy, and posterior instrumentation effectively managed the condition. Histopathological confirmation and a multidisciplinary team were essential for optimal outcomes. Long-term surveillance remains critical to detect local recurrence and lung metastasis, emphasising the need for ongoing vigilance in managing spinal GCTs.
